# Association of abnormal placental perfusion with the risk of male hypospadias: a hospital-based retrospective cohort study

**DOI:** 10.1186/s12884-020-03381-1

**Published:** 2020-11-07

**Authors:** Chen Zhu, Bin Zhang, Ting Peng, Ming-Qing Li, Yun-Yun Ren, Jiang-Nan Wu

**Affiliations:** 1 Department of Ultrasound, Obstetrics and Gynecology Hospital, Fudan University, 419 Fangxie Road, Shanghai, 200011 China; 2 Department of Obstetrics, Obstetrics and Gynecology Hospital, Fudan University, Shanghai, China; 3 Research institute of Obstetrics and Gynecology Hospital, Fudan University, Shanghai, China; 4 Shanghai Key Laboratory of Female Reproductive Endocrine-Related Diseases, Shanghai, China; 5 Department of Clinical Epidemiology, Obstetrics and Gynecology Hospital, Fudan University, 566 Fangxie Road, Shanghai, 200011 China

**Keywords:** Hypospadias, Abnormal placental perfusion, Uterine artery, Preeclampsia, Retrospective cohort study

## Abstract

**Background:**

The effect and extent of abnormal placental perfusion (APP) on the risk of male hypospadias are poorly understood. We compared the prevalence of male hypospadias in the offspring of women with APP and quantify the extent of the APP effect on the anomaly.

**Methods:**

A hospital-based retrospective analysis of births from 2012 to 2016 was conducted in 2018. Women of singleton pregnancy and male infants born to them were included (*N* = 21,447). A multivariate analysis was performed to compare the prevalence of male hypospadias in infants exposed to APP with those that were not exposed to APP.

**Results:**

Compared with the infants of women without APP, infants of women with APP showed an increased risk of male hypospadias (odds ratio, 2.40; 95% confidence interval, 1.09–5.29). The male hypospadias cumulative risk increased with the severity of APP. Infants exposed to severe APP had a significantly higher risk of male hypospadias than those without APP exposure (9.2 versus 1.7 per 1000 infants, *P* < 0.001). A path analysis indicated that 28.18–46.61% of the risk of hypospadias may be attributed to the effect of APP.

**Conclusions:**

Male hypospadias risk was associated with APP and increased with APP severity, as measured in the second trimester. APP had an important role in the development of the anomaly.

**Supplementary Information:**

The online version contains supplementary material available at 10.1186/s12884-020-03381-1.

## Background

Hypospadias are one of the most common birth defects in infants and have a substantial impact on childhood renal survival and the quality of life of affected children [1–3]. For example, infants with hypospadias may suffer from complications later in life, including a higher risk of testicular cancer and subfertility [4, 5], despite corrective surgery treatment. Furthermore, the prevalence of the anomaly in China is increasing [6]. According to the Report on the Prevention and Treatment of Birth Defects in China (2012), the prevalence of hypospadias has increased from 3.08 per 10,000 births in 1996 to 5.03 per 10,000 births in 2011 and ranks sixth among all birth defects in 2011 [7]. However, the causes of hypospadias are mostly unknown.

Placental dysfunction was considered a plausible candidate cause of congenital anomalies, such as hypospadias and congenital heart defects, because placental dysfunction may cause inadequate fetal hCG provision and intrauterine growth restriction and associate with angiogenic imbalance [3, 8, 9]. Placental dysfunction is often observed in pregnancies that are complicated by preeclampsia (PE), and markers of placental perfusion and function, such as uterine artery (UtA) pulsatility index (PI) and placental growth factor, therefore, have been employed to screen for PE [9–11], which was previously determined to be associated with the risk of cryptorchidism and hypospadias [12–15]. However, whether the cause of hypospadias is of placental origin or maternal genetic origin is still uncertain. Therefore, we conducted a retrospective cohort study to determine the relationship between abnormal placental perfusion (APP) and the risk of male hypospadias and quantify the extent of the effect of placental origin (indicated by APP, including UtA-PI, resistance index [RI], and early diastolic notching [EDN]) and maternal genetic origin (represented by PE, since PE has recently been observed as a maternal genetic origin disorder rather than the long-held viewpoint that PE is a placental disorder [16–18]).

## Methods

### Data and study design

We conducted a hospital-based retrospective study of pregnant women who received prenatal examinations at the hospital between April 2012 and August 2016 in Shanghai, China. Details of the design and methods have been described elsewhere [19]. Briefly, birth defect outcomes, including hypospadias, for infants reported in the Chinese Birth Defect Surveillance Network and in the Shanghai Perinatal Statistics Collection System were extracted and matched with hospital discharge reports for pregnant women that were compiled in the hospital electronic medical record system according to the unique identity card number of each mother. All women with singleton pregnancy of male fetuses were included in the study. The study was approved by the Ethics Committee of the Obstetrics and Gynecology Hospital of Fudan University (No. 2017–35, 2017–35-C1).

### Male hypospadias

Male hypospadias was diagnosed by clinicians according to the routine comprehensive physical examination for infants after delivery and was recorded in the medical information system [19]. Details of the anomaly were further collected to classify hypospadias as anterior hypospadias, middle hypospadias, and posterior hypospadias, as well as an unknown type when no details were available.

### Placental perfusion measurement and definition

Measurement of the UtA was recommended for pregnant women to screen for major organ malformations at 20 to 24 weeks gestation. All ultrasound scans were conducted using the GE Voluson-E6 and GE Voluson-E8 ultrasound devices (GE Healthcare, Zipf, Austria). Doppler measurements of the UtA were obtained by sonographers who have hold color Doppler large-scale instrument qualification certification according to standardized protocols [20]. Measurements for both UtA-PI and UtA-RI were calculated, and the presence of EDN was recorded. Therefore, there were six placental perfusion markers, including right and left UtA-PI, UtA-RI and EDN.

The quality of UtA-PI measurement, which was assessed by 95% limits of agreement using a randomly sample of 50 women, has shown that the measurement had a reasonable intra- and interoperator measurement repeatability (data not shown). Since the distribution of the UtA-PI and -RI values were nonnormal, the ninety-fifth percentile (P 95) of the values by gestational weeks at the time of the measurement was used to classify pregnant women into high (>P 95) UtA-PI and UtA-RI groups and normal (≤ P95) UtA-PI and UtA-RI groups. Data of P 95 for the four UtA indices by gestational weeks are shown in Table S1. UtA-EDN was diagnosed according to the ISUOG practice guidelines [20]. APP was defined by any abnormal values for the six markers (e.g., high right UtA-PI or -RI, high left UtA-PI or -RI, or positive for right or left UtA-EDN). Pregnant women who did not participate in uterine artery measurement were classified as the missing APP group.

### Covariates

PE is defined as the onset of hypertension and proteinuria after 20 weeks of gestation in women who were previously normotensive [21]. Pregnant women with PE were further identified as having severe PE if a continuous elevation of blood pressure (e.g., systolic blood pressure ≥ 160 mmHg, and/or diastolic blood pressure ≥ 110 mmHg) and/or urinary protein level (e.g., > 2.0 g/24 h), impaired maternal organ function (e.g., abnormal liver enzymes, dysopsia), or placental-fetal complications (e.g., fetal growth restriction, oligohydramnios) occurred [21]. Patients with eclampsia (an advanced form of PE that is characterized by convulsions of unexplained causes) were also included as severe PE (only 4 cases). Gestational hypertension (hypertension that develops after 20 weeks of gestation without proteinuria, 899 cases) and chronic hypertension with superimposed PE (new onset of proteinuria among women with preexisting hypertension, 308 cases) were classified as mild PE, as indicated in a previous study [22]. Women with chronic hypertension-complicated pregnancies (126 cases) were classified as no PE.

Potential confounders, including maternal age at delivery (≤ 24, 25–34, and ≥ 35 years), resident location (Shanghai and other provinces), parity (nulliparous and multiparous), assisted conception (yes and no), gestational diabetes mellitus (GDM, yes and no) and fetal gender (male and female), were extracted from the discharge reports. GDM was defined based on a 75 g oral glucose tolerance test [23]. Assisted conception pregnancies referred to pregnancies from in vitro fertilization or intracytoplasmic sperm injection and were self-reported from the pregnant women [19].

### Data analysis

The prevalence of hypospadias per 1000 infants and the corresponding 95% confidence interval (CI) for women with and without APP were calculated and compared using the chi-square test or Fisher’s exact test. Logistic regression models were created to estimate the crude odds ratios (ORs) and 95% CIs for hypospadias among infants who were exposed to APP compared with those without APP. Adjusted ORs and 95% CIs were evaluated after controlling for PE, maternal age at delivery, resident location, parity, mode of conception, GDM and fetal gender.

The relationships between variant APP (defined by any of the six markers) and the risk of male hypospadias were estimated. Pregnant women with APP were further grouped into mild (any 1 abnormal marker), moderate (any 2 abnormal marker) and severe (≥3 abnormal markers) APP subgroups according to the abnormal number of the six markers. The cumulative risk of male hypospadias across the three APP subgroups was calculated and compared with that in pregnant women without APP by the Kaplan-Meier curve method. A posteriori analysis was conducted in estimating the association between the risk of male hypospadias and APP classified by the situation of left UtA-RI and UtA-EDN, since it was accidentally determined that the two markers had stronger associations with the risk of male hypospadias than the other four markers.

Logistic regression analyses using path analysis models were conducted to decompose the total risk of male hypospadias into the effect of PE (presumed to be a maternal genetic effect) and the effect of APP (as a placental effect). We assumed two causal chains since the two factors were related. In path analysis, PE was set as a direct factor, and APP was set as an indirect factor, and vice versa [24]. The results of the path analyses were then combined according to the weight of the coefficient to explain the extent of the effect of APP if both PE and APP were directly and significantly associated with the risk of male hypospadias.

We restricted the sensitivity analyses to nulliparous women and those of natural conception because multiparity and assisted conception births may affect the severity of APP and may be associated with the risk of male hypospadias. Path analysis models were performed with Stata 12 software. All other statistical tests were conducted using IBM SPSS Statistics version 22.0. A two-sided *P* value < 0.05 was considered statistically significant.

## Results

### Basic characteristics of the study participants

A total of 52,047 pregnant women were involved in pregnancy examinations at the hospital between April 2012 and August 2016. Among these women, 21,447 (41.2%) pregnant women with a male fetus singleton pregnancy were included in this study. A total of 1861 pregnant women (8.7%) were classified as APP, and 1172 women (5.5%) were diagnosed with PE, including 212 women with severe PE (1.0%).

### Prevalence of male hypospadias

A total of 43 fetuses complicated with hypospadias were identified in this study, with an overall prevalence of male hypospadias of 2.5 per 1000 infants (95% CI, 1.8–3.3). The prevalence of hypospadias was higher for the infants of the women with APP than those of the women without APP (4.8 versus 1.7 per 1000 infants, *P* = .01) (Table 1). Most of the cases (76.7%, 33/43) involved the unknown type. Of the remaining 10 cases, 6 cases involved anterior hypospadias, 3 cases involved midline hypospadias and 1 case involved posterior hypospadias, which accounts for 60, 30 and 10%, respectively, of the cases.

**Table 1:** Characteristics and prevalence of male hypospadias

Characteristics		Male Hypospadias (*n* = 21,447, cases = 43)			
		Total no. of infants	No. of cases	Prevalence per 1000 infants (95% CI)	*P* value
Placental perfusion^a^					.03
Normal		14,186	24	1.7 (1.0–2.4)	
Abnormal		1861	9	4.8 (1.7–8.0)	.01^b^
NA		5400	10	1.9 (0.7–3.0)	.81
Preeclampsia					.001
No		20,275	36	1.8 (1.2–2.4)	
Yes	Mild	960	3	3.1 (0.0–6.7)	.26^b^
	Severe	212	4	18.9 (0.4–37.3)	.001^b^
	Any	1172	7	6.0 (1.6–10.4)	.008^b^
Maternal age at delivery (year)					.34^b^
≥ 35		2145	5	2.3 (0.3–4.4)	
25–34		18,224	38	2.1 (1.4–2.7)	
< 25		1078	0	0	
Residence					.59
Shanghai		16,536	35	2.1 (1.4–2.8)	
Other provinces		4884	8	1.6 (0.5–2.8)	
Parity					.14
Nulliparous		18,099	40	2.2 (1.5–2.9)	
Multiparous		3348	3	0.9 (0.0–1.9)	
Gestational diabetes mellitus					>.99^b^
No		19,616	40	2.0 (1.4–2.7)	
Yes		1831	3	1.6 (0.0–3.5)	
Assisted conception					.18^b^
No		21,061	41	1.9 (1.4–2.5)	
Yes		386	2	5.2 (0.0–12.3)	

*NA* APP data not available

^a^ Abnormal perfusion insufficiency was defined as any one abnormality of the six markers, which otherwise were normal; ^b^ *P* values for Fisher’s exact tests

### Association between APP and male hypospadias

Table 2 shows the association between APP and the risk of male hypospadias. Compared with the infants of the women without APP, the infants born to the women with APP were significantly associated with an increased risk of male hypospadias (OR, 2.40; 95% CI, 1.09–5.29). In the sensitivity analyses restricted to nulliparous and natural conception women, the association of APP with the risk of male hypospadias did not produce significantly different results (Table S2).

**Table 2:** Odds ratios of male hypospadias

Characteristics		Male hypospadias	
		Crude odds ratio (95% CI)	Adjusted odds ratio (95% CI)
Placental perfusion			
Normal		1.00	1.00
Abnormal ^a^		2.87 (1.33–6.18)	2.40 (1.09–5.29)
NA		1.10 (0.52–2.29)	1.12 (0.53–2.37)
Preeclampsia			
No		1.00	1.00
Yes	Mild	1.76 (0.54–5.73)	1.56 (0.47–5.11)
	Severe	10.81 (3.82–30.66)	7.75 (2.62–22.89)
Maternal age at delivery (years)			
≥ 35		1.00	1.00
25–34		0.89 (0.35–2.27)	0.74 (0.27–1.99)
< 25		–	–
Residence			
Shanghai		1.00	1.00
Other provinces		0.78 (0.36–1.67)	0.90 (0.41–1.96)
Parity			
Nulliparous		1.00	1.00
Multiparous		0.41 (0.13–1.31)	0.39 (0.11–1.33)
Gestational diabetes mellitus			
No		1.00	1.00
Yes		0.80 (0.25–2.60)	0.68 (0.21–2.23)
Assisted conception			
No		1.00	1.00
Yes		2.66 (0.64–11.02)	1.96 (0.45–8.55)

*NA* Not applicable

^a^ Abnormal placental perfusion was defined as any one abnormality of the six markers, which otherwise were normal

The association of the variants of APP with male hypospadias varied (Table 3). Women with a high left UtA-RI had an increased risk of having infants with hypospadias (OR, 2.86; 95% CI, 1.06–7.70) relative to those with normal RI. Women with left UtA-EDN were also associated with the risk of male hypospadias (OR, 5.33; 95% CI, 1.56–18.26) relative to those without left UtA-EDN.

**Table 3:** Variant of abnormal placental perfusion and odds ratio of male hypospadias

Variant of abnormal placental perfusion		Male hypospadias		
		No. of infants	No. of cases	Adjusted odds ratio (95% CI)
Right uterine artery				
PI	Normal (≤P95)	15,244	28	1.00
	High (>P95)	835	5	2.60 (0.97–6.98)
RI	Normal (≤P95)	15,348	29	1.00
	High (>P95)	731	4	2.34 (0.80–6.85)
EDN	No	15,942	32	1.00
	Yes	144	1	2.27 (0.29–17.65)
Left uterine artery				
PI	Normal (≤P95)	15,218	28	1.00
	High (>P95)	839	4	2.03 (0.68–5.99)
RI	Normal (≤P95)	15,267	27	1.00
	High (>P95)	790	5	**2.86 (1.06–7.70)**
EDN	No	15,859	30	1.00
	Yes	226	3	**5.33 (1.56–18.26)**
No. of insufficient markers				
0		14,186	24	1.00
1		663	3	2.50 (0.75–8.34)
2		872	3	1.79 (0.53–6.04)
3 and above		326	3	3.59 (0.99–12.95)
Left uterine artery RI and EDN				
Normal RI and without EDN		15,112	26	1.00
High RI or with EDN		866	6	**3.35 (1.34–8.38)**
High RI and with EDN		75	1	6.33 (0.68–41.70)
Any of high RI and/or with EDN		941	7	**3.53 (1.48–8.41)**

*PI* Pulsatility index, *RI* Resistance index, *EDN* Early diastolic notching

**Fig. 1:**
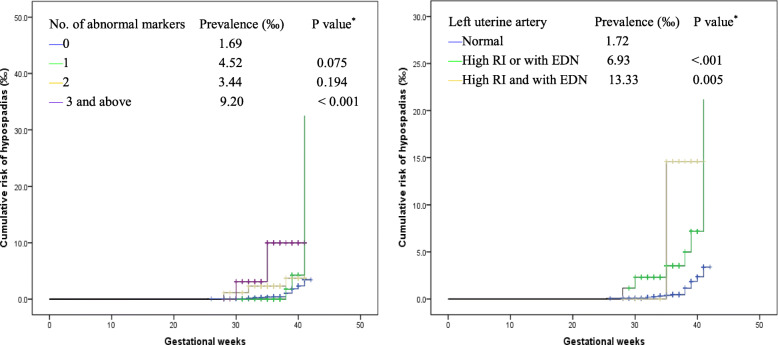
Cumulative risk of male hypospadias across the groups by the number of placental perfusion insufficiency markers and the situation of left uterine artery resistance index (RI) and early diastolic notching (EDN). ^*^ *P* values for the log rank (Mantel-Cox) method compared with the cumulative risk of male hypospadias in the reference group

The cumulative risk of male hypospadias among infants who were exposed to severe APP was higher than that among infants without APP exposure (9.20 versus 1.69 per 1000 infants, *P* < 0.001) (Fig. 1). However, no significant association between severe APP with male hypospadias was observed in the multivariate regression models (Table 3). Restricting APP to left UtA-RI and UtA-EDN led to much stronger associations with male hypospadias. Women who had both high RI and EDN had a higher cumulative risk of producing fetuses with hypospadias (13.33 vs 1.72 per 1000 infants, *P* = .005) than those with normal left UtA-RI and UtA-EDN, but there was no significant association with the risk of male hypospadias (Fig. 1, Table 3).

### Effect of APP on the risk of male hypospadias

Table 4 summarizes the results of the effect of APP on the risk of male hypospadias. Both PE and APP had a role in male hypospadias, with a total risk of 46.61% for male hypospadias, which may be attributed to the effect of APP in the total sample. Among women with severe PE and APP, the effect of APP on the risk of male hypospadias decreased to 28.18%, while neither mild PE nor APP was associated with the risk of male hypospadias among women with mild PE and APP (Table 4).

**Table 4:** Estimated size of the effect of abnormal placental perfusion on the risk of male hypospadias (%)^a^

No. of population	Direct factor (causal chain^b^)	Total effect			The effect of abnormal placental perfusion			
		Coef.	Standard Error	*P* value	Coef.	Standard Error	*P* value	Size of the total effect (%)
Infants (16051), any PE (874), APP (1861), anomalies (33)	Any PE (1)	1.30	0.49	.007	0.15	0.06	.009	11.54
	APP (2)	1.06	0.36	.003	0.95	0.34	.006	89.62
	Total (3)	2.36			1.10			46.61
Infants (15903), mild PE (726), APP (1792), anomalies (29)	Mild PE (1)	0.36	0.60	.54	0.04	0.05	.37	11.11
	APP (2)	0.50	0.56	.38	0.49	0.55	.37	98.00
	Total (3)	0.86			0.53			61.63
Infants (15325), severe PE (148), APP (1730), anomalies (31)	Severe PE (1)	2.68	0.60	<.001	0.30	0.16	.07	11.19
	APP (2)	1.01	0.35	.004	0.74	0.31	.02	73.27
	Total (3)	3.69			1.04			28.18

*PE* Preeclampsia, *APP* Abnormal placental perfusion

^a^Adjusted for maternal age at delivery (< 25, 25–34, or ≥ 35), residence (Shanghai or other provinces), parity (nulliparous or multiparous), gestational diabetes mellitus (yes or no), assisted conception (yes or no) and gender (male or female). ^b^Hypothetical causal chain: causal chain 1: PE was set as direct factor, and APP as indirect factor; causal chain 2: APP as direct factor and PE as indirect factor; causal chain 3: the weight of the coefficient of the effect of APP in chains 1 and 2 was combined when both of the causal chains (1 and 2) work (*p* value for the coeefficient <0.05)

## Discussion

In this retrospective cohort study of 21,447 single pregnancy Chinese women, the risk of fetal male hypospadias was associated with APP and increased with the severity of APP. Among the variants of APP, APP defined by a high left UtA-RI and positive EDN appeared to be most strongly associated with the risk of the anomaly. Compared with PE, APP had a mild-to-moderate role in the pathogenesis of male hypospadias, which accounts for 28.18–46.61% of the risk of male hypospadias. These findings confirmed the relationship between APP and the risk of male hypospadias and provided evidence for exploring the pathogenesis of the anomaly and the clinical screening and management of the disease.

### Context within previous literature

Early placental insufficiency was suggested as a plausible candidate cause for congenital anomalies in boys due to the vulnerable time window of genital morphogenesis in the first trimester [15]. Markers of placental insufficiency were considered linked with the risk of hypospadias. Placental weight and other placental characteristics and markers of placental insufficiency were examined in relation to hypospadias by Ghazarian AA and the colleagues using the Collaborative Perinatal Project [25]. However, except for placenta calcification, neither placental weight nor other placental characteristics (e.g., placental thickness, placental infarcts, amnion cell metaplasia, changes to the intervillous space, and intervillous thrombosis) were linked to the risk of hypospadias [25].

Impaired uteroplacental circulation is also a marker of placental insufficiency and has a central role in the pathogenesis of neonatal complications [26–29]. APP may be a consequent manifestation of defective or absent remodeling of the myometrial segment of the uteroplacental arteries [28–30] and accompanying placental dysfunction [31–33]. Placental insufficiency may cause inadequate fetal hCG provision, which is important for genitourinary development in the first trimester since hCG may stimulate fetal testicular steroidogenesis before the fetal pituitary-gonadal axis is established [3, 25, 34, 35]. Furthermore, the severity of APP was positively correlated with placental apoptosis and associated with an increased rate of preterm delivery and small-for-gestational age at birth [36]; both conditions are well-known and strong predictors of cryptorchidism and hypospadias [37, 38].

In this study, we discovered that both APP and PE were significantly associated with male hypospadias. These findings did not support that PE might only be a placental origin complication, which was consistent with a growing body of evidence that supports the role of maternal genetic origin in the pathogenesis of PE [16–18, 39]. We then introduced PE as a novel maternal factor in the pathogenesis model of male hypospadias and decomposed the total risk of male hypospadias into components of maternal and placental origin. We discovered that 53.39% (=1–46.61%) to 71.82% (=1–28.18%) of the risk of male hypospadias may be attributed to maternal (severe) PE. These findings were consistent with the results of previous studies, which revealed that genetic origin has a principal role in causing hypospadias and accounts for 57 to 77% of the phenotypic variability [3, 40–42]. APP, separately and/or through its association with PE, had a role in the risk of male hypospadias. The findings suggested a predominantly maternal genetic origin effect on the risk of male hypospadias [43].

### Implications of the study

Our findings help advance the understanding of the role of APP and PE in the pathogenesis of male hypospadias and have clinical value for management of the anomaly. First, the effect of PE on the risk of male hypospadias supports a predominantly maternal genetic origin-of-effect. Therefore, the prevention of hypospadias may predominantly depend on the ability to elucidate maternal genetic origin risk. Second, the association of APP with male hypospadias revealed that placental perfusion measurement may have a more valuable role in management of the disease, although the placental perfusion measurement might not have high predictive value for anomalies because of the fairly low prevalence of the outcomes. Thus, strategies for placental perfusion measurement should be optimized, including recognizing the necessity of measurement for obstetrics and recommending this procedure for all pregnant women. For people at high risk of male hypospadias, such as women with severe APP or at least one abnormality of left UtA-RI or UtA-EDN, further examinations of the fetal urogenital system are needed for the early detection and management of ongoing urogenital injury. These measures are particularly important in the context of the strict ban on sex determination by ultrasound and the rising prevalence rate of hypospadias in China.

### Limitations

There were some limitations in our study. First, the prevalence of infantile male hypospadias may be underestimated since 20% of the sample was lost to follow-up and possibly included women with miscarriages and terminated pregnancies. Fetuses born from these women were considered to have a high prevalence of birth defects [44, 45]. However, the association between APP and male hypospadias should not be affected by the underestimation. Second, 25.1% of the women did not participate in the placental perfusion measurement since the measurement was optional, and the infants born to these women might have an increased risk of infantile hypospadias, which suggests that women without APP measurements may have some characteristics associated with the disease. We were not able to estimate the potential impact of these characteristics on the associations between APP and hypospadias. However, we did not believe that these characteristics would affect the association between APP and hypospadias since the association was statistically significant in the logistic regression model, sensitivity analysis and path analysis. Third, details on pathology and phenotype were not available for further analysis and identification. For example, most of the cases (76.7%) were identified as the unknown type, and only one case underwent a pathological examination of the placenta (results showed APP, mild decidualitism, and placental villous stromal angioplasia with dilation). We were not able to link the findings and severity of the anomaly to understand the underlying pathophysiology. Furthermore, studies on the differences in relation to the risk of hypospadias across the six UtA indices were needed. Last, although the sample size was relatively large, this study was a single-center study of pregnant women in Shanghai, which might limit the generalizability of the findings.

## Conclusions

In this retrospective cohort study of single pregnancy Chinese women, we discovered that the risk of male hypospadias was associated with APP and increased with the severity of APP measured in the second trimester. APP had a mild-to-moderate effect on the risk of hypospadias compared with the role of PE.

## Supplementary Information

**Additional file 1:** **Table S1.** The ninety-fifth percentile (P95) of the values of the left and right uterine artery pulsatility index (PI) and resistance index (RI) by gestational week.

**Additional file 2:** **Table S2.** Odds ratio of male hypospadias in nulliparous and natural conception pregnant women.

## Data Availability

The datasets used in present study available from the corresponding author (wjnhmm@126.com) on reasonable request only.

## Abbreviations

APP: Abnormal placental perfusion
CI: Confidence interval
OR: Odds ratio
PE: Preeclampsia
PI: Pulsatility index
RI: Resistance index
UtA: Uterine artery
EDN: Early diastolic notching
