# Supplementary material for: Association of abnormal placental perfusion with the risk of male hypospadias: a hospital-based retrospective cohort study
Source: BMC Pregnancy Childbirth. 2020 Nov 7;20:673. doi: 10.1186/s12884-020-03381-1 (PMC7649004; doi:10.1186/s12884-020-03381-1)
Supplement: Supplementary file 1 — Additional file 1: Table S1. The ninety-fifth percentile (P95) of the values of the left and right uterine artery pulsatility index (PI) and resistance index (RI) by gestational week. [file 12884_2020_3381_MOESM1_ESM.docx]

Table S 1 The ninety-fifth percentile (P95) of the values of the left and right uterine artery pulsatility index (PI) and resistance index (RI) by gestational week

| Weeks at measurement | Right PI | Right RI | Left PI | Left RI |
| --- | --- | --- | --- | --- |
|  |  |  |  |  |
| 20 | 1.52 | 0.73 | 1.47 | 0.71 |
| 21 | 1.44 | 0.71 | 1.51 | 0.73 |
| 22 | 1.33 | 0.69 | 1.39 | 0.70 |
| 23 | 1.31 | 0.69 | 1.37 | 0.69 |
| 24 | 1.35 | 0.69 | 1.34 | 0.69 |
