# Supplementary material for: Association of abnormal placental perfusion with the risk of male hypospadias: a hospital-based retrospective cohort study
Source: BMC Pregnancy Childbirth. 2020 Nov 7;20:673. doi: 10.1186/s12884-020-03381-1 (PMC7649004; doi:10.1186/s12884-020-03381-1)
Supplement: Supplementary file 2 — Additional file 2: Table S2. Odds ratio of male hypospadias in nulliparous and natural conception pregnant women. [file 12884_2020_3381_MOESM2_ESM.docx]

Table S 2 Odds ratio of male hypospadias in nulliparous and natural conception pregnant women

| Characteristics | | Male hypospadias | |
| --- | --- | --- | --- |
|  |  | Nulliparous (n=18099, cases=40) | Natural conception (n=21063, cases=41) |
| Placental perfusion | |  |  |
| Normal | | 1.00 | 1.00 |
| Abnormal | | 2.57 (1.15-5.75) | 2.52 (1.13-5.65) |
| NA | | 1.14 (0.52-2.49) | 1.25 (0.59-2.66) |
| Preeclampsia | |  |  |
| No | | 1.00 | 1.00 |
| Yes | Mild | 1.70 (0.52-5.61) | 1.74 (0.53-5.72) |
|  | Severe | 8.20 (2.75-24.47) | 8.69 (2.92-25.87) |
| Maternal age at delivery (year) | |  |  |
| >=35 | | 1.00 | 1.00 |
| 25-34 | | 1.55 (0.36-6.65) | 0.80 (0.27-2.38) |
| <25 | | -- | -- |
| Residence | |  |  |
| Shanghai | | 1.00 | 1.00 |
| Other provinces | | 1.08 (0.50-2.35) | 0.96 (0.44-2.09) |
| Parity | |  |  |
| Nulliparous | | -- | 1.00 |
| Multiparous | | -- | 0.40 (0.12-1.37) |
| Gestational diabetes mellitus | |  |  |
| No | | 1.00 | 1.00 |
| Yes | | 0.78 (0.24-2.58) | 0.76 (0.23-2.49) |
| Assisted conception | |  |  |
| No | | 1.00 | -- |
| Yes | | 2.38 (0.54-10.40) | -- |
